# Anæsthetic Cases

**Published:** 1907-11-09

**Authors:** 


### Resident Medical Officers' Department.

[The Editor accepts no responsibility for any opinions expressed in these columns, which are freely open to all resident medical officers. Discussion and contributions are invited, and, if the latter are accepted, they will be paid for.]

### ANÆSTHETIC CASES.

A hospital resident, signing himself M. B., writes:—"A young woman, 20 years of age, developed symptoms pointing to an intracranial abscess (secondary to middle-ear mischief and mastoid disease), the exact site of which was not diagnosed. Examination of the lungs, heart and urine did not reveal anything to contraindicate the use of an anæsthetic. The patient was brought to the operating room on a stretcher, and the administration of a mixture of chloroform and ether in the proportion of $C_2E_3$ was commenced on a Schimmelbusch's mask with free admixture of air; that is to say, the mask was held at some distance from the face.

"In from two to three minutes after beginning the patient appeared to struggle slightly; the breathing was rather stertorous, the face became cyanosed, and finally respiration ceased. The mask was at once withdrawn, the mouth was opened, the tongue seized with forceps, and rhythmic traction practised. Artificial respiration was started, strychnine injected hypodermically, hot compresses applied to the præcordial region, etc., but without avail. Respiration appeared to be primarily affected, as the radial pulse could be felt after the untoward symptoms supervened. Less than half a drachm of the mixture had been dropped on the mask, and therefore considerably less must have been inhaled by the patient, as the mask was at no time firmly placed on the face.

"As regards the previous history of this case, the patient had within the preceding month undergone two operations for the mastoid disease. On these occasions the CE mixture was first employed, and then chloroform substituted. No difficulty had been experienced in giving the anæsthetic, and the condition of the patient during anæsthesia had never caused anxiety. The patient was not of a nervous temperament, and exhibited no signs of fear about having the anæsthetic.

"A post-mortem examination revealed a large cerebellar abscess on the left side. Almost all the cerebellar tissue on the affected side had been destroyed, and the cerebellum on that side was represented by a thin-walled abscess which had burst towards the base. All the other organs appeared healthy.

"Now we are here confronted with the questions, 'When did the abscess burst?' and 'What part did the anæsthetic play in the fatal result?' My own opinion is that the rupture took place at or about the time when the anæsthetic was started, and that the latter contributed only indirectly to the fatal termination. From the condition found 'post-mortem' I believe that any exertion on the part of the patient, such as sitting up suddenly in bed, might have caused the fragile wall to give way, but on this point I should be pleased to hear the opinion of others.

"In The Hospital of October 19 the question, 'Should house-officers be allowed to give anæsthetics?' is discussed. To the remarks there expressed probably no one can take exception, and it may be laid down that wherever possible the administration of the anæsthetic in all cases where difficulty is anticipated should be undertaken by a skilled person. This is a matter of comparative ease in the large London and Provincial hospitals, but what about the smaller hospitals, where there is no anæsthetist attached? Here the duty falls to the resident medical officer, and he may be called upon at any time to give the anæsthetic in a most difficult case. It therefore all the more behoves the medical schools to see that their students are compelled to study theoretically and practically anæsthetics and their administration."

The case of which "M. B." gives particulars is certainly one of interest, and it is very difficult to suppose that the chloroform could have directly brought about the fatal result. It is conceivable that rupture of a cerebellar abscess such as that described might be determined by the excitement stage of a chloroform induction; but it is again not easy to imagine that the small amount of drug administered here, spread over a period of two minutes or more, could produce enough spasm or excitement in a composed patient to bring about that result. On the whole, therefore, one feels inclined to agree with "M. B." in his interpretation of the train of events.

This incident of a fatality occurring in a patient who had recently been successfully anæsthetised under apparently similar conditions recalls a case which aroused some interest somewhat earlier in the century. A baby died, just before the commencement of an operation, at the hands of one of the best-known and most expert anæsthetists in London. About a month or five weeks before it had been successfully brought through a prolonged and severe operation, the administrator on that occasion being a house physician, and the anæsthetic used the same. The general condition of the patient was apparently far superior at the time of the second operation, as the first one had been performed for the relief of some acute abdominal mischief. If "M. B.'s" defence of the resident-medical-officer-anæsthetist required any support, the above case would be very much to the point. His remarks are in accordance with the policy which The Hospital has persistently advocated, for we are no believers in even metropolitan hospitals preventing their house officers from administering anæsthetics. In most hospitals the public suffers no detriment at all from that system; but if anywhere at any time it should so suffer, it becomes a bounden duty to speak plainly on the matter. While dealing with the subject, it must be mentioned that the fatalities during the past six months at Guy's Hospital number nine, not eleven as stated in our issue of October 19. The error does not invalidate the argument, and arose from erroneous reports in the daily press; but it was an error, and we apologise unreservedly for it.

